# Self-Reported Impact of Road Traffic Congestion on Persons Commuting to and From Work

**DOI:** 10.7759/cureus.104276

**Published:** 2026-02-26

**Authors:** Mandreker Bahall, Arvani Chaitram, Aravinda Deonarine, Arnalda Diptee, Arel Mieres, Anushka Moosay, Arianna Ramsahai, Ariana Sagam, George Legall

**Affiliations:** 1 Caribbean Centre for Health Systems Research and Development, University of the West Indies, St. Augustine Campus, Mount Hope, TTO; 2 School of Medicine, Faculty of Medical Sciences, University of the West Indies, St. Augustine Campus, Mount Hope, TTO

**Keywords:** anxiety, depression, leisure, mental health, productivity, road traffic congestion, stress

## Abstract

Background: Globally, road traffic congestion (RTC) represents a substantial public health problem contributing to negative psychological, social, and economic consequences, particularly among individuals experiencing RTC for prolonged periods of time. No studies have reported on the psychosocial impact of RTC locally within the 10-year period prior to the start of the study.

Aim: The aim of the study was to identify psychosocial/economic implications of RTC on selected adult travellers during peak traffic hours, with special emphasis on (1) stress, anxiety, and depression, (2) leisure and work-related experiences, and (3) loss of productivity.

Methods: Convenience sampling was used to select 385 study participants during a three-month period. The inclusion criteria were as follows: being at least 18 years of age, being a student or worker, commuting daily along a specified highway, and having experienced RTC during periods of peak traffic. Data were collected via face-to-face interviews at shopping centres. Variables measured included socio-demographics, self-reported depressive symptoms (Patient Health Questionnaire-2), anxiety (Generalised Anxiety Disorder-7), stress (Perceived Stress Scale-4), and selected social and economic indicators. IBM SPSS Statistics for Windows, V. 30.0 (IBM Corp., Armonk, NY, USA), was used for both descriptive and inferential statistical data analysis.

Results: A total of 275 usable responses (response rate of 71.4%) were analyzed. The sample was predominantly female (n = 163; 59.3%) and aged 18-27 years (n = 91; 33.1%). Participants were mainly from Central Trinidad (n = 79; 28.7%) and employed in the public sector (n= 104; 37.8%). The prevalence of severe anxiety was 9.8% (n = 27), severe depression 15.6% (n = 43), and moderate to severe stress 83.3% (n = 229). Spending 30 minutes or more in traffic was associated with a decrease in family time (p = 0.005). Nearly all participants were dissatisfied.

Conclusion: Participants reported significant levels of stress, anxiety, and depression, loss of leisure and family time, and loss of productive hours and overall dissatisfaction. Urgent attention is needed to expand road networks, stagger working hours, work from home, and enhance travel benefits.

## Introduction

Road traffic congestion (RTC) occurs when traffic demand exceeds road capacity, leading to reduced speeds, longer travel times, and vehicle queuing under non-free-flow conditions [1]. RTC is a global issue, affecting all areas of life including the physical, mental, social, and economic well-being of citizens [2]. A study by Phillips et al. reported that traffic congestion may be linked to higher levels of stress, fatigue, and depression among commuters, as well as diminished road safety [3]. In Trinidad and Tobago, persistent traffic congestion prompts commuters to adopt "averting behaviour", such as extra spending on food, childcare, communication, and after-work arrangements, leading to substantial additional personal costs [3]. Phillips et al. reported that these persons incurred an average additional monthly cost of TT$558, approximately TT$6696 annually [3]. An estimated TT$2.26 billion per year, which accounts for 1.37% of the gross domestic product (GDP) of Trinidad and Tobago, is the direct economic cost of traffic on the country [3]. Commuters in Trinidad and Tobago spend approximately 793 hours per year in traffic congestion, cumulatively 33 days, one month [3]. The direct and indirect consequences could include increased fuel usage, pollution, and lost productivity [4]. Time lost in traffic leads to critical time being taken away from leisure, family, and work [5].

Additionally, commuters experience physical problems such as back pain and dizziness, resulting from prolonged sitting in heavy traffic [6]. According to a study by Bou Samra et al., traffic congestion was also linked to significantly higher systolic and diastolic pressure in drivers; longer exposure was associated with greater increases [7]. In a cross-sectional study conducted in the United Arab Emirates (UAE), there was a negative relationship between commuting time and subjective well-being [8].

RTC has also led to major psychosocial issues such as stress [9], depression [10], anxiety [10], and social issues [9]. According to a study by Oweisana and Ordua, traffic congestion induces psychological stress for drivers, pedestrians, and commuters [9]. Studies conducted in the past have explored the economic burden and physical or mental effects of traffic exposure. No studies have reported on the psychosocial impact of RTC locally. Our study aims to determine the psychosocial/economic implications of RTC. More specifically, our objectives include the following: (1) stress, anxiety, and depression levels, (2) leisure and work-related problems, and (3) loss of productivity hours and its economic impact.

## Materials and methods

This was a cross-sectional study conducted between March 2025 and June 2025. Convenience sampling was used due to the inability to obtain a sampling frame. Potential participants were screened at shopping malls, as this setting was practical given time and resource constraints, provided high foot traffic of individuals willing to participate, and ensured interviewer safety. Responses were collected digitally after obtaining informed participant consent.

The inclusion criteria were as follows: being at least 18 years of age at the start of data collection period, commuting along a major highway that leads into North or Central Trinidad during peak hours (6:00-9:00 a.m. and 3:00-7:00 p.m.), having experienced traffic congestion to and/or from work in the city more than twice a week, and having spent at least half an hour in traffic daily. The exclusion criteria consisted of commuting for no other purpose but work or institutional education.

The data collection instrument was a 27-item questionnaire comprising the following: Socio-demographic (six items), Road Congestion Experience (nine items), Biopsychosocial and Economic Impact (11 items; Generalised Anxiety Disorder-7 (GAD-7) Scale), and Patient Health Questionnaire-2 (PHQ-2) and Abbreviated Perceived Stress Scale-4 (PSS-4) instructions and satisfaction level (one item). The self-developed questionnaire was pilot tested. Furthermore, the questionnaire utilised validated instruments. Physical symptoms were assessed by asking participants to indicate the frequency of congestion-related physical symptoms using options such as the following: "Yes, frequently"; "Yes, occasionally"; and "No, never". The GAD-7 scale was used to assess anxiety related to traffic congestion. The GAD-7 comprises seven items assessing anxiety symptoms (nervousness, ability to control worrying, excessive worrying, trouble relaxing, restlessness, annoyed/irritable, fearful), over the preceding two weeks. Items are rated on a 4-point Likert scale ranging from 0 (not at all) to 3 (nearly every day), yielding a total score between 0 and 21. Scores are categorised using established cut-off ranges, that is, 0-4 indicating minimal anxiety, 5-9 mild anxiety, 10-14 moderate anxiety, and 15-21 severe anxiety, with cut-off points at 5, 10, and 15, respectively. The scale demonstrates good reliability and validity [11]. Depressive symptoms were screened using the PHQ-2, which consists of two items assessing depressed mood and anhedonia over the past two weeks. Items are scored from 0 to 3, yielding a total score of 0-6, with scores ≥3 indicating clinically significant depressive symptoms. The PHQ-2 is a validated screening tool with good sensitivity and specificity [12]. Perceived stress was assessed using the four-item PSS-4, which evaluates the degree to which situations in one's life are appraised as stressful over the past month. Items were rated on a 4-point Likert scale ranging from 0 to 3, with positively worded items reverse-scored. Total scores ranged from 0 to 12, with higher scores indicating greater perceived stress. Scores ranged from mild (0-5), moderate (6-8), and severe (9-12). The PSS-4 has demonstrated acceptable reliability and validity in prior research [13]. Overall satisfaction with road traffic conditions was assessed using a single-item global rating scale, with responses ranging from 1 (most satisfied) to 10 (most dissatisfied). Single-item measures have demonstrated acceptable validity for assessing overall satisfaction [14]. All data were collected anonymously, securely stored in a password-protected Google Drive (Google LLC, Mountain View, CA, USA), accessible only to the research team, and retained for up to 12 months before permanent deletion.

The data were analyzed via IBM SPSS Statistics for Windows, V. 30.0 (IBM Corp., Armonk, NY, USA), using both descriptive and inferential methods. The latter included analysis of variance (ANOVA) and logistic regression. Results are presented in descriptive form as frequencies and percentages for categorical variables. Means with standard deviations and inferential statistics including chi-squared values and corresponding p-values (significant at <0.05) were used for selected variables.

Ethical approval for the study was obtained from the Campus Research Ethics Committee of the University of the West Indies, St. Augustine Campus (approval number: CREC-SA.2989/11/2024).

## Results

General 

Socio-Demographics

Out of 385 participants, 275 usable responses (response rate of 71.4%) were analyzed. The majority were female (n = 163; 59.3%), one-third (n = 91; 33.1%) were 18-27 years of age, and 3.3% (n = 9) were over 60. Most resided in Central Trinidad (n = 79; 28.7%), and 37.8% (n = 104) were employed in the public sector (Table 1). 

**Table 1:** Socio-demographic characteristics of the participants (total, n = 275)

Variable	n	%
Age (in years)		
18-27	91	33.1
28-37	66	24
38-47	64	23.3
48-60	45	16.4
Over 60	9	3.3
Gender		
Male	112	40.7
Female	163	59.3
Location of residence		
East	81	29.5
West	15	5.5
North	36	13.1
South	64	23.3
Central	79	28.7
Occupation		
Public sector worker	104	37.8
Other	171	62.2
Area of work		
East	35	12.7
West	6	2.2
North	156	56.7
South	78	28.4

Table 2 shows the frequency and percentage distribution of place of abode and place of work, with only 83 (30.2%) living and working in the same location.

**Table 2:** Residence and job location (total, n = 275)

	Job location: n (%)				
Residence	East	West	North	South	All
East	21 (7.6)	3 (1.1)	47 (17.1)	10 (3.6)	81 (26.5)
West	2 (0.7)	1 (0.4)	8 (2.9)	4 (1.5)	15 (5.5)
North	3 (1.1)	1 (0.4)	30 (10.9)	2 (0.7)	36 (13.1)
South	5 (1.8)	0 (0)	28 (10.2)	31 (11.3)	64 (23.3)
Central	4 (1.5)	1 (0.4)	43 (15.6)	31 (11.3)	79 (28.7)
All	35 (12.7)	6 (2.2)	156 (57.6)	78 (28)	-

RTC Experience

Approximately half (n = 141; 51.3%) of commuters reported experiencing traffic congestion more than five times per week, primarily during peak hours between 4:00 p.m. and 7:00 p.m. (n = 149; 54.2%). The mean daily travel time with traffic was reported as 1-2 hours (n = 80; 30.9%).

Psychological impact 

Self-Reported Anxiety

The reliability (internal consistency) of responses was excellent (Cronbach's alpha = 0.921). Close to half (n = 120; 43.6%) of the participants had a score of 0-1 out of 12 (Table 3).

**Table 3:** GAD-7 level distribution GAD-7: Generalised Anxiety Disorder-7

GAD-7 level	n	%
None to mild	120	43.6
Moderate	85	30.9
Moderately severe	43	15.6
Severe	27	9.8

**Figure 1:**
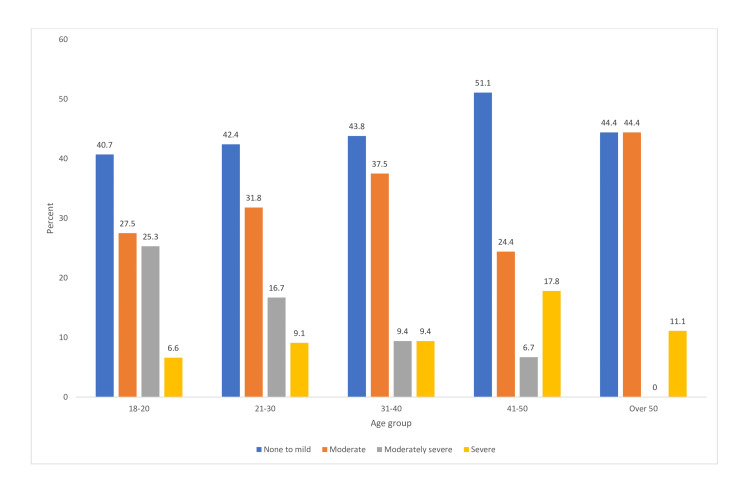
Anxiety levels by age group (n = 275)

Figure 1 shows the percentage distribution of levels of self-reported GAD-7 by age group. Over 40% of the participants in each age category self-reported none to mild anxiety; the prevalence of severe anxiety by age group ranged from 6% to 18%. 

Total score (out of 21) ranged from 0 (n = 34; 12.4%) to 21 (n = 10; 3.6%). The modal score was 7, as was the median. The mean was 7.1 (SD = 5.46).

Furthermore, ANOVA showed that the difference between the mean GAD-7 scores of participants who lived and worked in the same region and those who worked in a different region from the one in which they lived was statistically significant (p = 0.007).

Specifically, the mean anxiety score was greater among participants who worked outside of the region in which they lived than that of those who lived and worked in the same region.

The chi-squared analysis showed that whether or not participants lived and worked in the same region was associated with level of anxiety (chi-squared: 10.68; df = 3; p = 0.014), and ordinal logistic regression showed that it was also a predictor thereof (OR = 2.164; p = 0.001; 95% CI for OR (1.358, 3.445)). 

Depression

The prevalence of self-reported depression was 15.6% (n = 43). Scores ranged from 0 to 6, with a mean of 1.4 (SD = 1.43). The only significant difference between mean scores was that participants who lived and worked in the same region had a lower mean score than those whose work region was different from the region in which they resided (p = 0.006).

Furthermore, the chi-squared analysis showed a significant association between these two variables (chi-squared: 10.157; df = 1; p = 0.001), and binary logistic regression showed that where participants lived with respect to their place of work was a predictor of PHQ-2 depression (OR = 1.157; p = 0.001; 95% CI for OR (1.030, 1.263)).

Stress

Total PSS-4 scores ranged from 3 to 12 and were distributed by levels of severity as shown in Table 4, with the majority (n = 195; 70.9%) having moderate stress.

**Table 4:** Distribution of levels of stress

Stress	n	%
None	18	6.5
Mild	28	10.2
Moderate	195	70.9
Severe	34	12.4

The mean stress score was 7.0 (SD = 1. 97) and the mode was 8. ANOVA showed no significant differences between/among mean scores for any of the socio-demographic variables measured.

Bivariate correlations

Table 5 shows that each of the bivariate correlations among GAD-7, PHQ-2, and PSS-4 scores was positive and statistically significant.

**Table 5:** Bivariate correlations (p-value)

	Measure		
Measure	Generalised Anxiety Disorder-7	Patient Health Questionnaire-2	Perceived Stress Scale-4
Generalised Anxiety Disorder-7	1	0.680 (≤0.001)	0.276 (≤0.001)
Patient Health Questionnaire-2	0.680 (≤0.001)	1	0.277 (≤0.001)
Perceived Stress Scale-4	0.276 (≤0.001)	0.277 (≤0.001)	1

Impact on Physical and Mental Health

**Figure 2:**
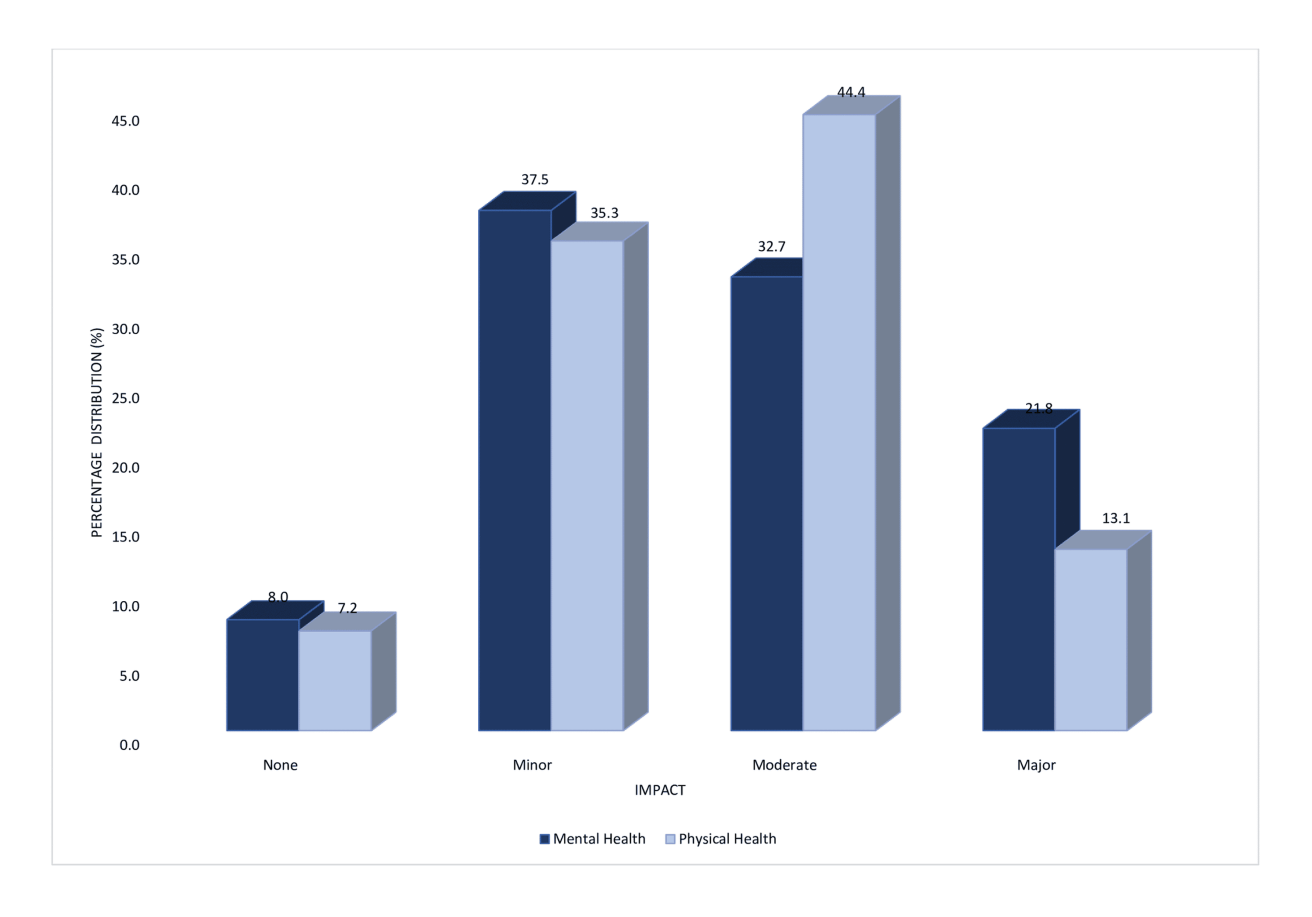
Self-reported impact of road traffic congestion on mental and physical health (n = 275)

Traffic congestion had a moderate effect which was greater on physical health than on their mental health, with the converse being true with respect to having a major effect (Figure 2).

The chi-squared analysis showed a significant association between the level of traffic congestion and physical health (χ² = 13.489; p = 0.009) and mental health (χ² = 10.804; p = 0.029).

Symptom experience

Physical 

Fatigue (n = 154; 56%) was the most reported physical symptom, followed by sleep deprivation (n = 108; 39.3%) (Table 6).

**Table 6:** Frequency of self-reported physical symptoms

Physical symptom	n	Percentage (%)
Fatigue	154	56
Sleep deprivation	108	39.3
Back pain	84	30.5
Headaches	83	30.2

Social well-being

Self-Reported Traffic-Related Outcomes

Patients experienced at least one of the following: loss of family time, loss of leisure time, and late for school/work due to traffic-related issues to and/or from work/school within the two-week period prior to being surveyed (Table 7).

**Table 7:** Self-reported traffic-related outcomes

Question	Response, n (%)			
Over the last two weeks, how often have you been bothered by the following problems? (Related to the traffic experienced)	Not at all	Several days	More than half the days	Nearly every day
Loss of family time	52 (18.9)	79 (28.7)	51 (18.5)	93 (33.8)
Loss of leisure time	36 (13.1)	78 (28.4)	55 (20)	106 (38.5)
Late for school/work	75 (27.3)	73 (26.5)	41 (14.9)	86 (31.3)

There was a significant association between age group and social activities: family, leisure, sports, and education (Table 8).

**Table 8:** Chi-squared association between age group and selected outcome

Outcome	Chi-squared (χ²) test	df	P-value
Loss of family time over the last two weeks	28.479	12	0.005
Loss of leisure time over the last two weeks	22.307	12	0.034
Loss of sporting time over the last two weeks	39.939	12	≤0.001
Less time for education over the last two weeks	25.787	12	0.012

Follow-up analysis (ordinal logistic regression) showed that age group was a predictor of "less time for education over the last two weeks - due to traffic congestion" only (Table 8).

Travel Work-Related Consequences 

Patients reported lateness (94.6%), absence (25.1%), sick days (26.9%), and missed appointments (38.5%) (Table 9). Penalties such as written warnings, reduced pay, loss of incentives, and promotion varied between 10% and 30% (Table 9).

**Table 9:** Distribution of occurrences experienced due to heavy traffic

Question	Response, n (%)			
Over the last two weeks, how often have you experienced the following problems? (Related to the traffic experienced)	Not at all	1-3 days	4-5 days	Nearly every day
Lateness	98 (25.6)	123 (44.7)	39 (14.2)	15 (5.5)
Absence	206 (74.9)	59 (21.5)	5 (1.8)	5 (1.8)
Sick days	201 (73.1)	0 (0)	70 (25.5)	4 (1.5)
Missed appointments	169 (61.5)	0 (0)	102 (37.1)	4 (1.5)

Work/Job-Related Penalties

Table 10 shows the frequency and percentage distribution of self-reported penalties for travel-related consequences of traffic congestion. 

**Table 10:** Penalties arising from travel-related consequences

Other consequences	Frequency		
	Never	Occasional	Frequent
Written warnings	189 (68.7)	76 (27.6)	10 (3.6)
Reduced pay for being late	243 (88.4)	25 (9.1)	7 (2.5)
Loss of punctuality incentives	200 (72.7)	63 (22.9)	12 (4.4)
Negative performance review	222 (80.7)	49 (17.8)	4 (1.5)
Loss of promotion	243 (88.4)	27 (9.8)	5 (1.8)

The percent reporting regular penalties was small, and the majority reported never having been penalised (Table 10). 

Economic impact

Eighty (29.1%) participants self-reported a severe increase in fuel usage due to prolonged periods of traffic congestion as the main economic impact.

Dissatisfaction

**Figure 3:**
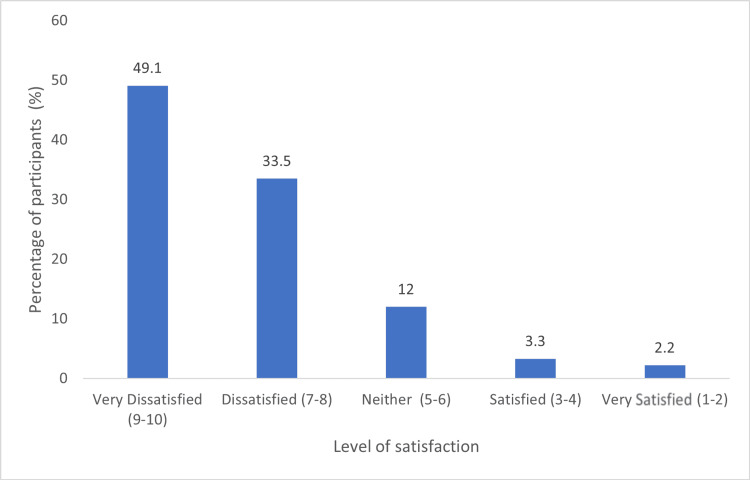
Level of satisfaction with travel to and from workplace due to traffic congestion (n = 275)

When asked "on a scale of 1-10, where 1 is least and 10 is most, how dissatisfied are you with the current RTC?", participants expressed high dissatisfaction. The summary statistics of responses were as follows: minimum 1 (n = 1; 0.4%), median 8, maximum 10 (n = 90; 32.7% ), mean (SD) 8.0 (2.09), and mode 10. Nearly half, 49.1% (n = 135), reported being "very dissatisfied" (Figure 3).

Table 11 shows selected summary statistics of total dissatisfaction scores.

**Table 11:** Total dissatisfaction score (out of 10)

Variable	Age group	n	Summary statistics				
			Minimum	Median	Maximum	Mean	SD
Gender							
Female	18-27	48	1.0	8.5	10.0	7.9	2.43
	28-37	39	5.0	8.0	10.0	8.1	1.71
	38-47	43	2.0	8.0	10.0	8.2	1.91
	48-60	28	1.0	10.0	10.0	8.2	2.72
	60+	5	5.0	6.0	10.0	7.0	2.00
	All females	163	1.0	8.0	10.0	8.0	2.17
Male	18-27	43	1.0	8.0	10.0	7.3	2.31
	28-37	27	3.0	8.0	10.0	8.2	1.83
	38-47	21	5.0	9.0	10.0	8.3	1.49
	48-60	17	6.0	9.0	10.0	8.9	1.36
	60+	4	6.0	9.5	10.0	8.8	1.89
	All males	112	1.0	8.0	10.0	8.0	1.98
All	18-27	91	1.0	8.0	10.0	7.6	2.38
	28-37	66	3.0	8.0	10.0	8.2	1.75
	38-47	64	2.0	8.0	10.0	8.2	1.77
	48-60	45	1.0	10.0	10.0	8.4	2.31
	60+	9	5.0	8.0	10.0	7.8	2.05
	All ages	275	1.0	8.0	10.0	8.0	2.09

There were no associations between satisfaction and age (p = 0.180) or sex (p = 0.913) of participants (Table 11). 

## Discussion

Socio-demographics

The results revealed that almost one-third of commuters were individuals aged 18-27 years (n = 91; 33.1%) and more than half were female (n = 163; 59.3%) and within the public sector (n = 104; 37.8%). A similar study by Phillips et al. found that 56% (n = 148) of respondents were female, 53% (n = 140) were aged 31-45, and 56% (n = 148) and 10% (n = 26) classified their occupation as "professionals" and "clerks", respectively [3].

Road traffic features

Approximately half (n = 141; 51.3%) of commuters reported experiencing traffic congestion more than five times per week, primarily during peak hours between 4:00 p.m. and 7:00 p.m. (n = 149; 54.2%). The mean daily travel time with traffic was reported as 1-2 hours (n = 80; 30.9%), which compares favourably with a 2017 study with urban Canadians, who spent approximately 56-101 minutes in traffic daily [15]. The majority (n = 254; 92.4%) relied on private vehicles. At the time of the study, the majority of participants (n = 178; 64.7%) were living outside the region in which they worked. Phillips et al. reported that 83% (n = 219) commuted via private vehicles [3].

Biopsychosocial factors and their associations

Self-reported moderate to severe stress was the most frequently reported psychological outcome, affecting 83.3% (n= 229) of participants. This finding is consistent with a 2022 study conducted in Port Harcourt Metropolis, Nigeria (Oweisana and Ordua), which demonstrated a significant relationship between prolonged time spent in traffic and increased stress levels [9]. In the present study, this may be attributed to Trinidad's road network infrastructure being outpaced by urban expansion and a major increase in vehicles, thus promoting a stressful driving environment [3]. The prevalence of depression was 15.6% (n = 43). A 2023 Taiwanese study found that with increased exposure to road traffic noise, the prevalence of depression also increased [16]. Severe self-reported anxiety was more prevalent among participants who worked outside the region in which they lived and was more common among persons aged 48-60 years. The prevalence is comparable to that of a 2023 study in Korea, which observed that levels of anxiety increased with prolonged commute times [10]. However, this contrasts with a French study in 2023, which concluded that anxiety levels had a negative correlation with age [17]. 

The most common physical symptoms experienced were fatigue (n = 154; 56%), sleep deprivation (39.3%), and back pain (30.5%). These findings were similar to a 2021 study conducted in Dhaka, Bangladesh, which found that tiredness and low back pain were most common due to prolonged periods of sitting [18].

The results (Figure 2) illustrated that commuters noticed greater effects on their physical health with 44.4% (n = 122) noting a moderate impact, while 32.7% (n = 90) noted a similar impact on their mental health. This compared well with the 2017 study in Sharjah, Malaysia, in which respondents claimed that long driving hours led to adverse physical effects [6]. Traffic congestion was associated with physical and mental health outcomes (p = 0.009 and p = 0.029, respectively).

Participants' social lives were also affected. There was a loss of time spent with family and for relaxation. Furthermore, prolonged traffic exposure resulted in less time for daily activities. Between 15% and 30% of participants were also disciplined in their workplace with only a few (1-5%) frequently. These findings are consistent with evidence reported by Clark et al., who found that longer commute times were associated with less leisure and job time, along with poorer mental health [19].

Commuters felt that fuel costs (n = 80; 29.1%) had severely increased. This is similar to the findings of the 2022 socioeconomic impact study in Bangladesh, which credited the stop-and-go operation of the vehicles during congestion to increased fuel consumption and, by extension, cost [4].

Our results showed that close to half were very dissatisfied, less than 10% were very satisfied, and one-third were dissatisfied. Dissatisfaction levels were also reported by Boneo and Townsend in Port of Spain, Trinidad, in 2023, and similar results were obtained, whereby participants were strongly dissatisfied with congestion and lack of accessibility within the nation's capital city [20].

Limitations of the methodology

Data collection was conducted within a limited time frame in a few geographic areas and might have compromised a proper sample representation. The study was too small for subgroup analysis. A larger sample size may be required in more venues to allow greater participation and increased coverage. Patients' recall may be compromised since participants were interviewed in malls or shopping centres where they would be out of reach of the hustle of the road congestion.

Recommendations

Efforts must be made to expand road networks, stagger working hours, work from home, improve travel, and improve productivity while travelling. Additionally, upgrading and expanding road infrastructure can alleviate traffic bottlenecks and improve traffic flow. Overall, RTC represents an important public health concern, warranting targeted transport and workplace policy interventions. There is a need to further research this area and address similar issues with a more appropriate sample to compare validity.

## Conclusions

Participants reported significant levels of stress, anxiety, and depression, loss of leisure and family time, and loss of productive hours. There is a need for a complete change in travel to minimise health problems and optimise productivity.
